# The 3114: A new professional helpline to swing the French suicide prevention in a new paradigm

**DOI:** 10.1192/j.eurpsy.2022.2318

**Published:** 2022-10-07

**Authors:** C.-E. Notredame, M. Wathelet, M. Morgiève, P. Grandgenèvre, C. Debien, C. Mannoni, N. Pauwels, F. Ducrocq, E. Leaune, P. Binder, S. Berrouiguet, M. Walter, P. Courtet, G. Vaiva, P. Thomas

**Affiliations:** 1 Psychiatry Department, CHU Lille, 59000 Lille, France; 2 PSY Lab, Lille Neuroscience & Cognition Centre, INSERM U1172, Lille University, 59000 Lille, France; 3 Groupement d’Étude et de Prévention du Suicide, 86280 Saint-Benoît, France; 4 Fédération Régionale de Recherche en Psychiatrie et Santé Mentale des Hauts-de-France, 59350 Saint-André, France; 5 Centre National de Ressources et Résilience pour les psychotraumatismes (Cn2r), 59000 Lille, France; 6 CERMES3, CNRS, INSERM, University of Paris, 75006 Paris, France; 7 Center for Suicide Prevention, Centre Hospitalier le Vinatier, 69500 Bron, France; 8 Department of General Medicine, Medicine and Pharmacy, University of Poitiers, 86000 Poitiers, France; 9 LaTIM, INSERM, UMR1101, 29200 Brest, France; 10 Psychiatry Department, CHU Brest, 29609 Brest, France; 11 EA 7479 SPURBO, West Brittany University, 29238 Brest, France; 12 Department of Emergency Psychiatry and Acute Care, CHU Montpellier, 34000 Montpellier, France; 13 IGF, University of Montpellier, CNRS, INSERM, 34000 Montpellier, France

**Keywords:** Helpline, suicide, suicide prevention

## Abstract

Helpline services have been identified as an important component of suicide prevention strategies. While the Covid-19 pandemic has raised major concerns about severe and longstanding mental health consequences, the French Ministry of Health and Prevention has recently decided to implement a national professional helpline dedicated to suicide prevention. The 3114 has been launched on October 1, 2021. Accessible 24/7 from any point of the national territory, it offers remote assistance to individuals in distress or worried about a close one, professionals, and bereaved persons. Spread in regional call centers, medically supervised nurses and psychologists provide callers with listening, evaluation, intervention (including the possible dispatch of a rescue team), and whenever needed, referral to adapted services. At the same time, the “3114 centers” contribute to the implementation of the regional suicide prevention strategies by stimulating the development of actions, promoting resources, monitoring at-risk events, and collaborating with professional and associative stakeholders. From a public health perspective, the inception of the 3114 has settled the conditions for a new paradigm in the French suicide prevention strategy. By dedicating specific resources to promote and organize interactions between stakeholders, it supports a major shift from the juxtaposition of efficient but segregated actions to the creation of an integrated prevention system. Embedded to the project, multidisciplinary and multilevel research will be carried out to evaluate the implementation, impact, and transferability of the 3114 model, conceived both as a helpline and territorial prevention strategy.

Despite a trend decline since the beginning of the century, suicide rates in Europe remain among the highest of WHO regions [1]. In the past 10 years, France has consistently belonged to the half of most impacted European countries [2]. In 2017, 8,214 French individuals died by suicide [3]. Lately, strong concerns have emerged about the fact that mental health consequences of the Covid-19 pandemic may translate into longstanding increases in suicide rates worldwide [4], and more specifically in Europe [5]. In France, alarming indicators tend to support this hypothesis. For instance, from 2018–2019 to 2021, admissions to the ED for suicidal ideations and suicide attempt in 11–17 years old adolescents—especially girls—have increased by about 130 and 30%, respectively [6, 7]. First figures from 2022 suggest even more concerning patterns, with increases in suicidal behaviors rates extending to older age classes [7].

In line with the recommendation of the European Psychiatric Association that “unprecedented times are calling for unprecedented efforts” [5], the French government has recently decided to complete its national strategy for suicide prevention by implementing a universal professional helpline service. Helplines are one of the historical pillars of suicide prevention. In the 1950s, Samaritans have inaugurated in Great Britain a massive spread of telephone help services across the world. Based on an important—although methodological fragile—corpus of evidence supporting their efficacy [8], helplines are now recommended as an important selective component of suicide prevention strategies [9].

In France, local and national suicide-prevention helplines have burgeoned since the 1960s. Almost all consisted of trained volunteers providing distressed callers a nondirective listening. Recently, national instances remarked the lack of a complementary professional service able to provide distressed individuals more proactive and evidenced-based remote evaluation and preventive interventions. More specifically, the absence of referral to mental health professionals was identified as a missed opportunity to promote and facilitate access to care. In addition, the only way of getting immediate help in case of an acute suicidal crisis was by calling the general emergency numbers, with nonoptimal responses due to shortage of specialized skills. Finally, numerous suicide prevention actors called for a reliable, visible, and unified national professional helpline as a resource to promote in the community, broadcast in the media, spread on the web, and display on hotspots.

To fulfill the gap, the 3114, French national helpline for suicide prevention, has been launched by the French minister of health on October 1, 2021. Freely accessible 24 h a day and 7 days a week from any part of the country including overseas territories, it targets any individual needing assistance with suicidal issues, which encompasses (a) distressed persons, ranging from emerging suicidal ideations to acute crisis, (b) persons worried about a close one, (c) professionals needing information or advice, and (d) persons bereaved by suicide.

The development and implementation of the 3114 are coordinated by the “National pole” of the 3114, a federative organization hosted by the University Hospital of Lille which closely collaborates with various experts, institutions, and associations. The French Ministry of Health and Prevention, which funds the project, steers its general orientation, and ensures its conformity with the current regulatory framework.

From the operational point of view, the French national helpline has a decentralized organization. The regional spread of “3114 centers” (1–2 per region) allows for proximal responses and accurate knowledge of local resources. These centers host the “respondents” of the 3114, that is, nurses and psychologists placed under the supervision of a senior psychiatrist. Respondents as well as supervisors received a specifically designed training based on an expert consensus and evidence-grounded practices. The remote professional help they offer consists of: (a) nonjudgmental and empathic listening, with the objective of rapidly creating, reinforcing, and maintaining an alliance with the caller; (b) a targeted clinical evaluation, which comprises an estimation of the suicide risk, the identification of salient psychopathological signs and symptoms and an assessment of the psychosocial context; (c) a strategical intervention, which may include counseling, motivational reinforcement and/or problem-solving. The objective is to bring a first relief, commit the caller to the caring process and mobilize his/her personal and external resources; and (d) referral to adapted sanitary (i.e., general practitioner, psychiatric services, or emergency room), welfare, social or associative resources, with the possibility to program a follow-up contact. A tight collaboration with emergency services allows for dispatching a rescue team whenever the caller is estimated in immediate danger. Also, in a global care perspective, respondents are supported by a social worker when financial needs, housing problems, educative precarity, or difficulties in accessing social rights are identified.

Beyond this distant help activity, the 3114 regional centers also have a territorial mission. Thanks to social workers and network managers, they contribute promoting available suicide prevention actions, federating stakeholders, monitoring the occurrence of at-risk events (e.g., hot spots), reducing territorial and social inequalities, and stimulating the development of innovations under the pilotage of Regional Health Agencies. The structure, missions, and actions of the 3114 are summarized in Figure 1.

The launch of the 3114 has been supported by an intensive communication campaign with the diffusion of print and digital flyers, collaboration with journalists, organization of webinars with local actors, promotion by national institutions, and active social media publications. A specifically designed website (www.3114.fr) has been developed to provide the general population with various specified information, advice, and resources about suicidal behaviors and prevention.

The implementation of the 3114 represents a turning point for suicide prevention in France. In 8 months of existence, it already received about 90,000 calls ranging from early prevention for mild distress without suicidal ideation to acute suicidal crisis needing immediate rescue. Complementary to associative services, it ensures tailored responses based on tight articulations with healthcare service, which has been pinpointed as a key condition to reduce suicide rates [10]. The implantation of the 3114 regional centers also represents a strong opportunity to put in synergy prevention actors and actions in accordance with local needs, thus helping to concretely translate the WHO recommendations for a multilevel (i.e., combining targeted, indicated and universal actions), multisectoral (i.e., base of the cooperation of the sanitary, associative, social and community sectors), multidisciplinary (i.e., appealing to various disciplines such as biomedicine, epidemiology, and sociology) and multimodal (i.e., combining modalities of actions such as communication, healthcare, and training) prevention strategy. In that, the 3114 swings French prevention into a new paradigm, from animating multiple efficient but juxtaposed actions (such as the brief contact intervention system VigilanS [11]) to shaping a coherent territorialized prevention ensemble.

Importantly, the 3114 is still at an inception stage. Developments are ongoing to reach a mature, stabilized system: opening of the last call centers, consolidation of the digital and technical solutions, implementation of a continued training for responders and supervisors, improvement of work conditions, reinforcement of intercenters collaborations, strengthening of the access for the undeserved and people living with a disability, promotion of deeper territorial anchoring, stepping-up of networking activities and leveling-up of the communication strategy for greater local and national visibility. On top of these routine developments, the National Pole and the French Ministry of Solidarity and Heath have already anticipated major evolutions for the 3114. Among those, the opening of a 24/7 online chat is intended to broaden the spectrum of reached populations both in terms of sociodemographic (especially toward youth) and clinical characteristics. In a further effort to support the development of a national digital prevention strategy, the 3114 may also open a social network channel, so as people can contact respondents directly from their daily communication apps. Because such innovation is highly sensitive from a technological, regulatory, and ethical point of view, the National Pole will rely on insights from the Elios project, a national randomized control trial assessing the efficacy of allowing suicidal young adults to get a direct contact with web clinicians through Instagram and Messenger [12].

Given its key role in the French public health strategy for suicide prevention, it is crucial to evaluate the relevance and effectiveness of the 3114, both as a hotline and a territorial prevention actor. With respect to distal indicators, the national all-at-once implementation represents both an opportunity and a hurdle to impute observed changes to the service: on the one hand, it allows for observing the time trends of suicidal behaviors precisely before and after the launching date, on the other hand, it prevents any comparison with equivalent noncovered territory. However, as noticed by Hoffberg et al., evaluation of crisis lines is a multifaceted area covering much more than the reduction of suicidal behavior [8]. To meet this challenge, the National Pole intends to broaden its research interest and implement several studies based on collected data and metadata. The following protocols are already planned: (a) description of calls and callers’ characteristics producing follow-up indicators, (b) medico-economic evaluation of the cost of suicidal behavior and comparison to the period preceding the opening of the service, (c) embedded monitoring of the respondents’ mental health—the final aim being the production of recommendations to promote better quality of life for the professionals, (d) sociological observations that will help drawing lessons from the 3114 about the sociocultural determinants, political constrains and contextual leverages or obstacles to be taken in account when implementing such a broad and complex prevention project, and (e) assessment of the opinion and representations of the service users as well as upstream and downstream partners in order to improve the acceptability of the 3114. Of note, systematically collected data will be also made available to external research teams upon application. Overall, the idea is to shed light on the relevance of the 3114 model, but also on the transferability of this model to other contexts and countries.

## Data Availability

The data about the number of calls received by the 3114 are available upon request to recherche@3114.fr.

**Figure 1.**
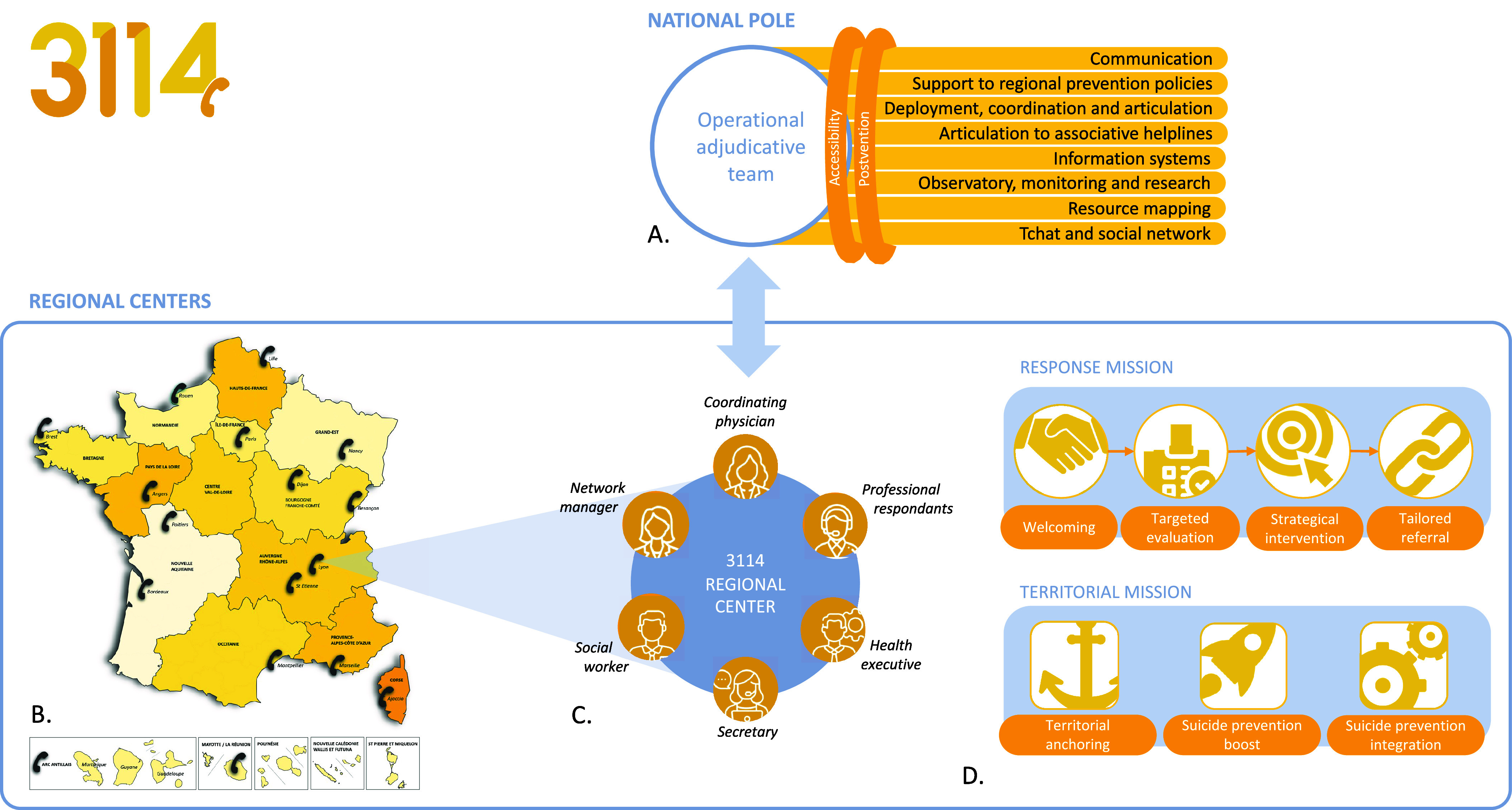
Structure, missions, and actions of the 3114. (A) The 3114 National Pole is composed of eight strategic poles (horizontal light-yellow lines) and two transversal thematic axis (dark-yellow rings), coordinated by an operational adjudicative team (gray circle). (B) Seventeen regional centers (black phones) spread over the French territory hold the 3114 missions. Each regional center is assigned an area of operation. (C) The 3114 regional centers are composed of clinical, operational, and supervisory professionals. (D) Each 3114 regional center has both a response and a territorial mission. As regards to territorial missions, territorial anchoring comprises articulations with healthcare and community resources, participation in health democracy organizations, cooperation with health policymakers, and mapping of the sociodemographic and epidemiological indicators. Suicide prevention boost comprises contribution to surveillance of suicidal behaviors, provision of information and alerts to stakeholders, and identification of prevention opportunities. Suicide prevention integration comprises operational articulation between prevention actions and systems, promotion, facilitation of collaboration between stakeholders, mobilization of the community, and resource sharing. Structure, missions, and actions of the 3114. (A) The 3114 National Pole is composed of eight strategic poles (horizontal light-yellow lines) and two transversal thematic axis (dark-yellow rings), coordinated by an operational adjudicative team (gray circle). (B) Seventeen regional centers (black phones) spread over the French territory hold the 3114 missions. Each regional center is assigned an area of operation. (C) The 3114 regional centers are composed of clinical, operational, and supervisory professionals. (D) Each 3114 regional center has both a response and a territorial mission. As regards to territorial missions, territorial anchoring comprises articulations with healthcare and community resources, participation in health democracy organizations, cooperation with health policymakers, and mapping of the sociodemographic and epidemiological indicators. Suicide prevention boost comprises contribution to surveillance of suicidal behaviors, provision of information and alerts to stakeholders, and identification of prevention opportunities. Suicide prevention integration comprises operational articulation between prevention actions and systems, promotion, facilitation of collaboration between stakeholders, mobilization of the community, and resource sharing.
